# Mapping the relative risk of weight disorders in children and adolescents across provinces of Iran: the CASPIAN-V study

**DOI:** 10.34172/hpp.2020.37

**Published:** 2020-07-12

**Authors:** Marzieh Nasr, Mohammadali Pourmirzaei, Mohammad Esmaeil Motlagh, Ramin Heshmat, Mostafa Qorbani, Roya Kelishadi

**Affiliations:** ^1^Child Growth and Development Research Center, Research Institute for Primordial Prevention of Non-Communicable Disease, Isfahan University of Medical Sciences, Isfahan, Iran; ^2^Pediatrics Department, Child Growth and Development Research Center, Research Institute for Primordial Prevention of Non-Communicable Disease, Isfahan University of Medical Sciences, Isfahan, Iran; ^3^Pediatrics Department, Ahvaz Jundishapur University of Medical Sciences, Ahvaz, Iran; ^4^Chronic Diseases Research Center, Endocrinology and Metabolism Population Sciences Institute, Tehran University of Medical Sciences, Tehran, Iran; ^5^Non-communicable Diseases Research Center, Alborz University of Medical Sciences, Karaj, Iran

**Keywords:** Geographic mapping, Relative risk, Underweight, Overweight, Children

## Abstract

**Background:** This study aimed to find possible spatial variation in children’s weight disorders and in predicting the spatial distribution.

**Methods:** The study population of this ecological study consisted of 7-18-year-old students living in 30 provinces of Iran. We used Besag, York and Mollie (BYM) model, a Bayesian model, to study the relative risk (RR) of underweight and excess weight (overweight and obese). The model was fitted to data using OpenBUGS (3.2.1) software.

**Results:** The highest RR of underweight was found in southeastern provinces. Whereas, the highest RR of excess weight was documented in northern, northwestern and capital provinces.Sistan-Balouchestan (RR=1.973; Bayesian confidence interval [BCI]: 1.682, 2.289), Hormozgan(RR=1.482; BCI: 1.239, 1.749), South Khorasan (RR=1.422; BCI: 1.18, 1.687) and Kerman(RR=1.413; BCI: 1.18, 1.669) had the highest RR of underweight. Mazandaran (RR=1.366; BCI:1.172,1.581), Gilan (RR=1.346; BCI: 1.15,1.562), Tehran (RR=1.271; BCI: 1.086,1.472) and Alborz (RR=1.268; BCI: 1.079,1.475) provinces are high risk regions for excess weight.

**Conclusion:** The significant variations in geographical distribution of weight disorders are because of various sociodemographic and ethnic differences. The current findings should be considered in health policy making in different regions of the country.

## Introduction

The current status of children’s health in each nation displays the future of that nation. One of the most important health indicators is, in this regard, having appropriate weight in childhood and adolescence. Indeed, the consequences of either underweight or overweight can negatively affect the physical growth and psychological development of children.^1^ In addition, muscle weakness, delayed puberty, and low work productivity are negative outcomes of underweight.^2^ On the other hand, overweight children are more likely to become overweight adults, which indicates high risks for vascular disease, diabetes mellitus type 2, metabolic diseases, and mental disorders.^3^ Moreover, some studies link fatness to significantly lower quality of life.^4,5^ Morbidity and mortality in adulthood are possible consequence of pediatric obesity.^6^

Body mass index (BMI) is used to determine childhood overweight and obesity. Overweight is defined as a BMI at or above the 85th percentile and below the 95th percentile for children and teens of the same age and sex. Obesity is a BMI at or above the 95th percentile for children and teens of the same age and sex.^7^

Iran is the world’s 18th most populated country, whose population is estimated at 81.91 million in 2019. Its territory spans 1 648 195 km^2^ (636 372 m^2^), making it the 17th largest in the world.^8^

Childhood obesity is one of the serious problems in Iran with an increasing trend putting children and adolescents at risk for poor health. According to an Iranian national study, the prevalence of overweight and obesity was reported to be 9.7% and 11.9%, respectively.^9^ In a meta-analysis study in 2016, the prevalence of obesity in Iranian population below the age of 18 was estimated as 6.1%.^10^ In another meta-analysis study in 2014, the overall prevalence of obesity and overweight were estimated to be about 5.1% and 10.8%, respectively.^11^ However, these figures had great spatial variation over the 31 provinces of the country. In addition, different age and sex groups had large variations in the prevalence of obesity and overweight.^12^ This study is interested in finding possible spatial variation in children’s disorder weight and predicting its spatial distribution, considering the effect of age and sex. Although, many studies have been conducted on this subject in Iran, few of them have virtually focused on the spatial distribution.^13-18^

Disease mapping, the analysis of geographical variation in rates of disease, can identify areas of unusually high risk so that action may be taken. Nowadays, disease mapping methods are widely used in many countries to model the geographical distribution of common diseases such as cancer, premature mortality due to cardiovascular disease and obesity, malaria, health inequalities, public health improvements and mortality.^19-23^ In addition, these methods are used to formulate etiological hypotheses and finally provide a map of disease risk in a region to allow better resource allocation and risk assessment.^24,25^ In fact, one of the main needs of health specialists is the regulation of health promotion programs. In other words, the awareness of the geographical distribution of the relative risk (RR) of weight disorders in children and adolescents can help prepare programs to prevent many future health problems including adult obesity, type 2 diabetes and heart disease. Focusing on health promotion and disease prevention programs in the pediatric age group can provide effective strategies for reducing further population-level burden of disease.

Using traditional methods based on the raw or standardized data was been very common in the last decade. One of the most common methods used by many researchers to estimate the RR of disease is standardized mortality ratio (SMR) method. Although, it has several disadvantages. Firstly, the mean and variance of SMR are dependent upon expected numbers. And secondly, in regions where there are not any cases of disease, the SMR is zero. Today, the use of disease mapping methods has led to controlling the problem of SMR and creating the high-precision maps.^26^ On the other hand, previous studies in Iran only have focused on a small area. While awareness of disease trend can help to allocate the resources equitably.

The aim of the present study is mapping of RR of Iranian children and adolescent weight disorders in the provinces of Iran based on a national school-based program. Thus the Besag, York and Mollie (BYM) model has been used with the Bayesian approach.

## Materials and Methods

### Study design

This study is an ecological study at provinces of Iran level, based on the CASPIAN-V study, which was conducted in 30 provinces of Iran at both urban and rural levels in 2015.^27^

### Participants

Students aged 7–18 years were the target population of the study. Students were selected using multistage, stratified cluster sampling method. The sample size was 14 400 students at the national level, i.e., 480 students in each province.^27^ Data of all these students were included in the current study without any restrictions.

### Measurements

Weight and height were measured under standard protocols using calibrated instruments BMI was calculated by dividing weight (kg) by height squared (m^2^). In order to categorize BMI, the WHO growth curve was used in which BMI was classified into underweight (BMI <5th percentile), normal weight (BMI between 5th and 85th percentiles), overweight (BMI between 85th and 95th percentiles), and obese (BMI ≥85th–95th percentiles).^7^

### Statistical analysis

*BYM Model:* BYM model was used to study the incidence of underweight and excess weight (overweight and obese) RR. In this model, {*Y*_i_*,i = 1,...,I* } and {*E*_i_*,i = 1,...,I* } represent the number of observed and expected cases for province *i* , respectively, where *E*_i_*= n*_i_*(∑Y*_i_*)/( ∑n*_i_*)* , i = 1, 2,…, I (n is the number of sample size of province).

It is assumed that *Y*_i_ has Poisson distribution with the rate of *μ*_i_*= θ*_i_*E*_i_, where *θ*_i_ represents RR for province *i* . This model considers two sources of changes to justify the heterogeneity the rate of incidence in every region. Then, *θ*_i_, the RR of diseases in *i* region, is modeled as:

*logθ*_i_ *=* *α +* *u*_i_ *+ v*_i_

Where *u*_i_ and *v*_i_ represent non-structural and structural heterogeneity, respectively.^24,28^

This model was fitted using OpenBUGS 3.2.1 (rev 781).^29^ Convergence was checked by using Brooks-Gelman-Robin plots. Significance tests for parameters were done by the use of Bayesian confidence intervals (BCI) which are equivalent to *P* value. All the maps were provided using ArcGIS (10.4.1).^30^

## Results

We intend to provide a map of children and adolescent weight disorders in Iran. We applied BYM model with Bayesian approach. Table 1 provides a descriptive statistic of data. Overall, 16.1% were underweight, and 20.8% had excess weight (overweight and obese).

**Figure 1:**
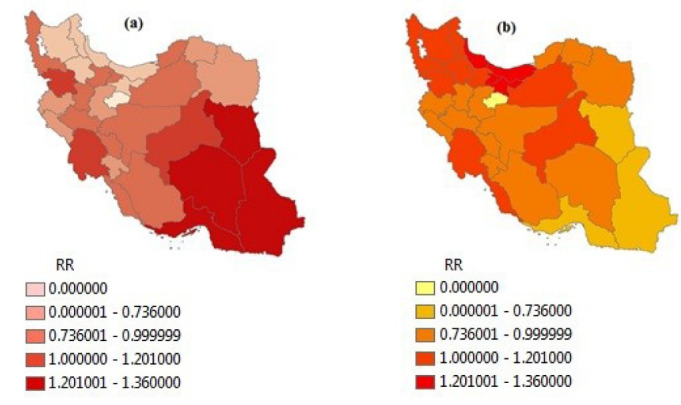
The map of relative risk of (a) underweight and (b) excess weight (overweight and obese) in Iranian children and adolescence by using BYM model; RR<0.7: low risk, 0.7≤RR<1: medium risk, 1≤RR<1.2: high risk, RR≥1.2: extremely high risk.

Table 2 shows the RR, observed (Oi) and expected (Ei) numbers of underweight and excess weight cases in provinces of Iran, based on BYM model. Figures 1a and 1b show the map of RR of underweight and excess weight from BYM model, respectively. Sistan-Balouchestan (RR = 1.973), Hormozgan (RR = 1.482), South Khorasan (RR = 1.422) and Kerman (RR = 1.413) have the highest RR of Underweight as shown in Figure 1a. West-Azarbayejan, Mazandaran, Gilan, Tehran, Zanjan and Ardebil have the least RR of Underweight. West-Azarbayejan with RR = 0.499 has the least risk. According to Figure 1b, Mazandaran (RR = 1.366), Gilan (RR = 1.346), Tehran (RR = 1.271) and Alborz (RR = 1.268) provinces are high risk regions for excess weight.

**Figure 2:**
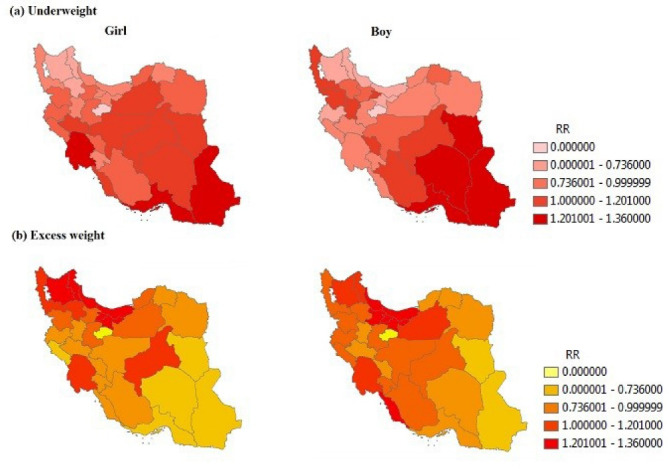
The maps of relative risk of (a) underweight and (b) excess weight, separated by sex in Iranian children and adolescence by using BYM model; RR<0.7: low risk, 0.7≤RR<1: medium risk, 1≤RR<1.2: high risk, RR≥1.2: extremely high risk.

Figure 2 illustrates the findings of the study for girls and boys, separately. According to Figure 2a, Khuzestan (RR = 1.55), Hormozgan (RR = 1.425) and Sistan-Balouchestan (RR = 1.55) provinces have the highest RR of underweight for girls and South Khorasan (1.44), Kerman (RR = 1.40), Hormozgan (RR = 1.50) and Sistan-Balouchestan (RR = 2.19) provinces have the highest RR of underweight for boys. The most important finding observed is that girls of Khuzestan province are so affected by underweight.

According to Figure 2b, Mazandaran (RR = 1.28), Gilan (RR = 1.36), Ardebil (RR = 1.17), East-Azarbayjan (RR = 1.19), Tehran (RR = 1.19) and Alborz (RR = 1.17) provinces have the highest RR of excess weight for girls and Mazandaran (RR = 1.32), Gilan (RR = 1.20), Qazvin (RR = 1.23), Tehran (RR = 1.28), Alborz (RR *=* 1.24) and Bushehr (*RR =* 1.38) provinces have highest RR of excess weight for boys. The most important finding seen in these maps is that girls in Ardebil and East-Azarbayjan provinces and boys in Qazvin and Bushehr provinces are affected by excess weight. These findings have not been shown by maps of overall sex in Figure 1.

**Figure 3:**
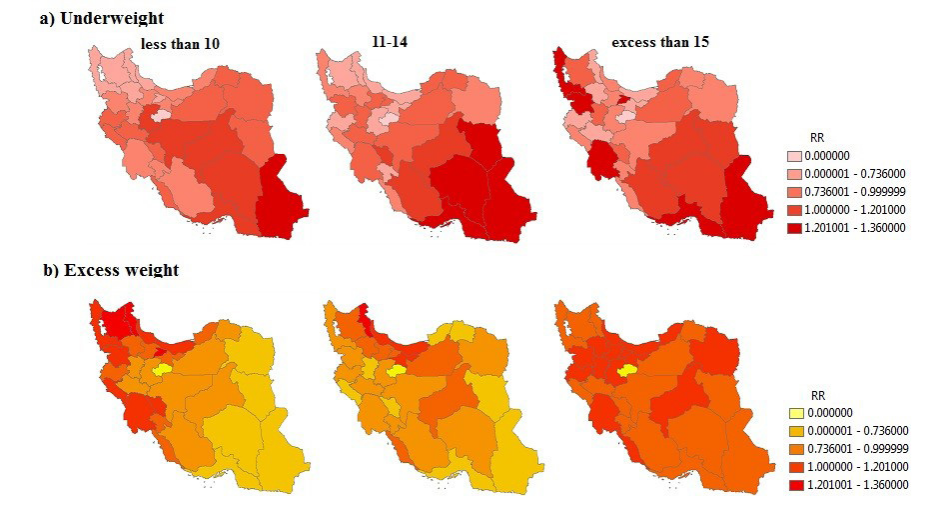
The maps of relative risk of (a) underweight and (b) excess weight, separated by age groups in Iranian children and adolescence by using BYM model; RR<0.7: low risk, 0.7≤RR<1: medium risk, 1≤RR<1.2: high risk, RR≥1.2: extremely high risk.

The results obtained from the separate age groups are shown in Figure 3. According to Figure 3a, Sistan-Balouchestan province has the highest RR of underweight in less than 10 years old (RR = 1.76) and 11-14 (RR = 2.04) age groups, respectively. West-Azarbayejan (RR = 2.43) and Khuzestan (RR = 2.17) provinces have the highest RR in the over 15-year-old age group.

According to Figure 3b, Alborz province (RR = 1.49) has the highest RR in the less than 10-year-old age group. Ardebil province (RR = 3.41) has the highest RR in the 11-14-year-old age group. However, no significant difference was seen among the provinces in the age group of those aged 15 or over.

## Discussion

The current study presents the spatial pattern of RR of weight disorders by sex and different age groups in provinces of Iran by using Bayesian BYM model in a large representative sample of Iranian students. BYM model in comparison with traditional methods such as SMR, provides more reliable estimates and more precision maps by using the required information from spatial correlation among various areas. Bayesian approach to disease mapping includes prior knowledge about the variation in disease rates, as well observed cases in each region to provide a more precise spatial pattern of disease.^24,25^

It was revealed that the highest RR of excess weight (overweight and obesity) occurred in northern, northwestern and capital provinces including Gilan, Mazandaran, Alborz, and Tehran. This finding supports previous research in this field,^13,14,18,31^ whereas the highest RR of underweight occurred in southeastern provinces including Sistan-Balouchestan, Hormozgan, south Khorasan, and Kerman. These results seem to be consistent with the other research done in these areas.^15-17^

Although, according to a systematic review study, the prevalence of obesity in Iran was not very diverse,^14^ the finding is in agreement with some published studies which showed the prevalence of obesity among women and girls in northern and northwestern provinces.^31-33^

One unanticipated finding was related to mapping of underweight in the third age group (≥15). In contrast to previous finding that West Azarbayejan was one of the areas with high risk of excess weight, it is known as an area at risk of underweight in the present study. The reason for this is not clear but it may be a high tendency to consider thinness as the preferred body image style, especially in women.^34^

The existence of significant differences in geographical areas can be due to differences in lifestyle, food habits, health facilities, socio-economic status, and even genetics. In order to find out more precise information on risk factors in high-risk areas, studies should be conducted at an individual level.

In the present study, the target population was divided to two sex groups and three age groups, separately. Therefore, more accurate diagnosis of the at-risk population has been achieved.

However, our study had some limitations. Data was at provinces level and it would be better to study children’s disorder weight distribution on smaller regions such as counties.

### Implications for practice and policy making

1. Using the results of this study to recognize at-risk regions and population.
2. Using the results of this study to implement health promotion and prevention program in at-risk regions.

### Future direction

It is recommended to conduct a similar study for a longer period of time in more recent years. In addition, exploring the association between socio-environmental status and human developing index with disorder weight would be provide a new understanding of socioeconomic inequality.

## Conclusion

Awareness of the geographical distribution of the RR of weight disorders in children and adolescents can contribute to the development of health promotion programs in the future. Taking childhood weight disorder, especially obesity, as one of the most serious problems of nations, establishing health promotion programs in schools including changing unhealthy eating habits and increasing physically activities can be effective. It is well documented that, training healthy behaviors to prevent chronic disease is easier during childhood and adolescence. Therefore, it is important to mention that performing these programs is only possible when parents would have close cooperation with school personnel.

## Ethical approval

This study was performed using data of the CASPIAN-V study to assess the geographical distribution of collected data. Data management and analysis were performed without access to patients’ information. All personal information was kept confidential.

## Competing interests

None to declare.

## Funding

This study was conducted as part of a national surveillance program.

## Authors’ contributions

RK, MP, MEM, RH and MO have made substantial contributions to conception, design and conducting. MN has been made contributions to dada analyses and drafting the study. RK has been made substantial contributions to critically review the manuscript for important intellectual content, and has given final approval of the version to be published.

## Acknowledgments

Authors are thankful from the large team working on this large nationwide project.

**Table 1:** Frequency (n), BMI mean and Standard Deviation (SD) of underweight and excess weight cases according to sex and age (year)

	**Total**		**Sex**				**Age**					
			**Girl**		**Boy**		**≤10**		**11-14**		**≥ 15**	
	**n**	**Mean (SD)**	**n**	**Mean (SD)**	**n**	**Mean (SD)**	**n**	**Mean (SD)**	**n**	**Mean (SD)**	**n**	**Mean (SD)**
Underweight	2279	13.97(1.54)	1030	13.70(1.52)	1249	14.19(1.51)	841	12.82(0.94)	915	14.07(1.13)	523	15.64(1.30)
Excess weight	2945	24.44(5.70)	1428	24.43(4.36)	1517	24.45(6.72)	923	21.46(6.24)	1242	24.52(5.02)	780	27.85(3.79)

**Table 2:** Relative risk (RR), 95% Bayesian confidence interval of RR (BCI), observed (O_i_) and expected (E_i_) number of Underweight and Excesss weight cases in provinces of Iran, based on Besag, York and Mollie (BYM) model

**Provinces**	**Underweight**				**Excess weight**			
	**RR**_i_	**BCI**	**O**_i_	**E**_i_	**RR**_i_	**BCI**	**O**_i_	**E**_i_
Ardabil	0.677	(0.51,0.86)	112	96.764	1.161	(0.98,1.37)	45	65.621
West Azarbayjan	1.066	(0.86,1.30)	98	96.970	1.025	(0.86,1.22)	85	75.041
East Azarbayjan	0.499	(0.36,0.65)	104	84.797	1.193	(1.00,1.42)	31	74.881
Bushehr	0.968	(0.77,1.18)	124	99.033	1.170	(0.99,1.38)	72	76.637
Chaharmahal Bakhtiari	1.056	(0.86,1.28)	77	97.795	0.845	(0.70,1.01)	82	76.637
Isfahan	1.070	(0.88,1.28)	95	99.033	0.961	(0.82,1.13)	84	76.637
Fars	1.015	(0.82,1.22)	96	99.033	0.960	(0.81,1.13)	75	76.637
Qazvin	0.953	(0.77,1.15)	105	99.033	1.088	(0.93,1.27)	78	76.637
Gilan	0.712	(0.55,0.89)	139	99.033	1.346	(1.15,1.57)	54	76.637
Golestan	0.948	(0.76,1.16)	84	99.033	0.894	(0.74,1.07)	73	75.52
Hamedan	0.994	(0.81,1.20)	78	99.033	0.864	(0.72,1.02)	79	76.637
Hormozgan	1.482	(1.24,1.75)	67	99.033	0.736	(0.60,0.89)	116	76.478
Ilam	0.857	(0.68,1.06)	87	99.033	0.904	(0.75,1.08)	64	76.637
Kerman	1.413	(1.18,1.67)	90	99.033	0.869	(0.73,1.04)	109	76.637
Kermanshah	0.782	(0.61,0.96)	96	99.033	0.959	(0.81,1.14)	56	76.637
South Khorasan	1.422	(1.18,1.68)	62	99.033	0.694	(0.57,0.84)	106	73.444
Razavi Khorasan	0.879	(0.70,1.08)	93	99.033	0.926	(0.78,1.1)	63	76.637
North Khorasan	0.920	(0.73,1.13)	77	99.239	0.820	(0.67,0.99)	70	76.637
Khuzestan	1.176	(0.97,1.41)	120	99.033	1.135	(0.97,1.33)	95	76.637
Kohgiluyeh & Boyerahmad	0.795	(0.63 , 0.98)	94	99.033	0.965	(0.82,1.14)	55	76.637
Kurdestan	1.135	(0.93,1.37)	101	99.033	1.009	(0.85,1.19)	92	76.797
Lorestan	0.988	(0.80,1.19)	85	97.589	0.902	(0.76,1.06)	76	76.158
Markazi	0.871	(0.70,1.06)	94	99.033	0.983	(0.83,1.16)	65	76.637
Mazandaran	0.612	(0.47,0.77)	144	98.414	1.366	(1.18,1.59)	38	76.637
Alborz	1.044	(0.84,1.27)	129	99.033	1.268	(1.08,1.48)	84	75.679
Semnan	1.051	(0.86,1.27)	106	99.033	1.051	(0.90,1.23)	85	76.637
Sistan & Blouchestan	1.973	(1.67,2.29)	49	98.827	0.580	(0.46,0.72)	156	76.637
Tehran	0.685	(0.53,0.85)	131	99.033	1.271	(1.09,1.48)	47	76.637
Yazd	1.245	(1.03,1.48)	112	99.033	1.052	(0.90,1.24)	98	76.637
Zanjan	0.674	(0.52,0.83)	96	94.907	1.039	(0.87,1.22)	46	76.637
